# Effects of Different Anxiety Levels on the Behavioral Patternings Investigated through T-pattern Analysis in Wistar Rats Tested in the Hole-Board Apparatus

**DOI:** 10.3390/brainsci11060714

**Published:** 2021-05-27

**Authors:** Maurizio Casarrubea, Giuseppe Di Giovanni, Giuseppe Crescimanno

**Affiliations:** 1 Laboratory of Behavioral Physiology, Department of Biomedicine, Neuroscience and Advanced Diagnostics (Bi.N.D.), Human Physiology Section “Giuseppe Pagano”, University of Palermo, 90134 Palermo, Italy; giuseppe.crescimanno@unipa.it; 2 Interdepartmental Center for Science Technology (C.I.T.C.), University of Palermo, 90141 Palermo, Italy; 3 Laboratory of Neurophysiology, Department of Physiology and Biochemistry, Faculty of Medicine and Surgery, University of Malta, MSD2080 Msida, Malta; giuseppe.digiovanni@um.edu.mt; 4 Neuroscience Division, School of Biosciences, Cardiff University, Cardiff CF10 3AX, UK

**Keywords:** Hole-Board, Head-Dip, Edge-Sniff, T-pattern analysis, diazepam, FG7142

## Abstract

The Hole-Board is an ethologically based tool for investigating the anxiety-related behavior of rats following manipulation of the central anxiety level. The present paper aims to assess behavioral patterning following pharmacological manipulation of emotional assets in Wistar rats tested in this experimental apparatus. For this purpose, the behavior of three groups of rats injected with saline, diazepam or FG7142 was evaluated using conventional quantitative and multivariate T-pattern analyses. The results demonstrate that quantitative analyses of individual components of the behavior, disjointed from the comprehensive behavioral structure, are of narrow utility in the understanding of the subject’s emotional condition. Among the components of the behavioral repertoire in rodents tested in the Hole-Board, Edge-Sniff and Head-Dip represent the most significant ones to rate anxiety level. They are characterized by a strong bivariate relationship and are also firmly part of the behavioral architecture, as revealed by the T-pattern analysis (TPA), a multivariate technique able to detect significant relationships among behavioral events over time. Edge-Sniff → Head-Dip sequences, in particular, are greatly influenced by the level of anxiety: barely detectable in control animals, they completely disappear in subjects with a reduced level of anxiety and are present in almost 25% of the total of T-patterns detected in subjects whose anxiety level increased.

## 1. Introduction

Generalized anxiety disorder, separation anxiety disorder, panic disorder, and agoraphobia, just to name a few, represent different neuropsychiatric conditions encompassed in the wide-ranging perimeter of anxiety disorders [1]. Although each form of anxiety disorder is characterized by specific features, based on the definitions and descriptions provided by the Diagnostic and Statistical Manual of Mental Disorders (DSM-V), all anxiety disorders share features of excessive fear and anxiety and related behavioral disturbances [1]. Common behaviors in patients with anxiety disorders may be represented by the avoidance of specific activities, people, objects, places, situations, etc., believed to produce anxiety and, not infrequently, the excessive consumption of drugs or alcohol. These maladaptive behaviors, chronically maintained, have deleterious consequences, such as a decline of interpersonal relationships, deterioration of job-related activities and an ineluctable “corrosion” of life quality. Not surprisingly, the social–economic burden of anxiety disorders is no less than catastrophic. Last but not least, these problems, during the last decades have been observed to be in a constant increase [2,3,4,5,6]. In this context, it is not difficult to guess why pre-clinical studies using animal models, aimed at a better understanding of the behavioral features of anxiety disorders, have an extremely important role [7]. In several studies, we utilized the Hole-Board (HB) apparatus and various ethologically based approaches to investigate the anxiety-related behavior of rats following manipulation of the central anxiety level. The HB is, basically, an open field with a given number of ground holes where the rodent can insert its head. The HB is a simple tool to study various exploration- and anxiety-related components of the behavioral repertoire in rodents [8,9]. Rather than evaluating quantitative aspects of a single parameter, simply considering it a mirror of the subject’s emotional drive, we employed structural analyses of behavior by evaluating whether and how the relationships between the various elements of the behavioral repertoire changed as a result of reductions or increases in the anxiety level. Among these relationships, the patterning between Head-Dip and Edge-Sniff and, in particular, the so-called Head-Dip/Edge-Sniff ratio, proved to be of reliable use as an indicator of the rat’s anxiety-related behavior with particular emphasis on the emotional condition [8,9]. A coherent investigation in this direction is to assess whether the patterning between Head-Dip and Edge-Sniff maintains only the aforementioned bivariate value or, on the other hand, if (and eventually how) these two important elements of the behavioral repertoire of the rat in HB are contextualized in the structure of the behavior.

Through the analysis of T-patterns (TPA), it is possible to perform detailed structural analysis of the behavior, evaluating existing temporal relationships between events over time. The detection process of T-patterns, within a given observation period, is performed using a software specifically developed for this purpose known as Theme (Patternvision, Reykjavik, Iceland). Using such an approach, it is possible to detect sequences of behaviors characterized by statistically significant relationships among the events in sequence. 

However, the sequential features of T-patterns and their hierarchical and multi-ordinal characteristics are very different from other techniques, such as hierarchical clustering methods or lag sequential analysis. On this subject, a more detailed description of concepts and theories can be found in various papers from the creator of this multivariate approach [10,11,12,13,14,15,16,17,18].

T-patterns are widely and advantageously used in numerous research areas and they can offer very useful results in studies involving both human and animal subjects. For example, using TPA, it is possible to study neuropsychiatric diseases [19,20,21,22], route-tracing stereotypies in mice [23], the interactions between humans and animals [24] or between humans and artificial agents [25], interactions between hormones and behavior [26], feeding behavior in rodents [27], and the behavior in a model of Tourette’s syndrome [28] and in a model of Parkinson’s disease [29]; TPA has been used also to study movement and behavioral disorders mainly in human subjects but also in animals [30]. Finally, and importantly, with numerous studies available, T-pattern analysis has demonstrated its usefulness and affordability also in the study of rodent anxiety and anxiety-related behaviors [31,32,33,34,35,36,37,38,39]. The present study aims to apply the T-pattern analysis to evaluate whether Head-Dip and Edge-Sniff, under different anxiety levels, show a broader patterning than the simple bivariate relationship and, if so, to evaluate how this patterning is contextualized in the behavioral structure. To this purpose, two different compounds, very well known to act on different directions of the emotional profile, namely diazepam and FG7142 [8,9,40], were used. Shedding light on this aspect could allow improving the usefulness of the Head-Dip/Edge-Sniff patterning by adding the temporal dimension and, therefore, a furthermore sensitive and reliable tool to study, in rodents, anxiety-related behavior and the behavioral effects of anxiety level manipulation. 

## 2. Materials and Methods

### 2.1. Experimental Apparatus

**Figure 1:**
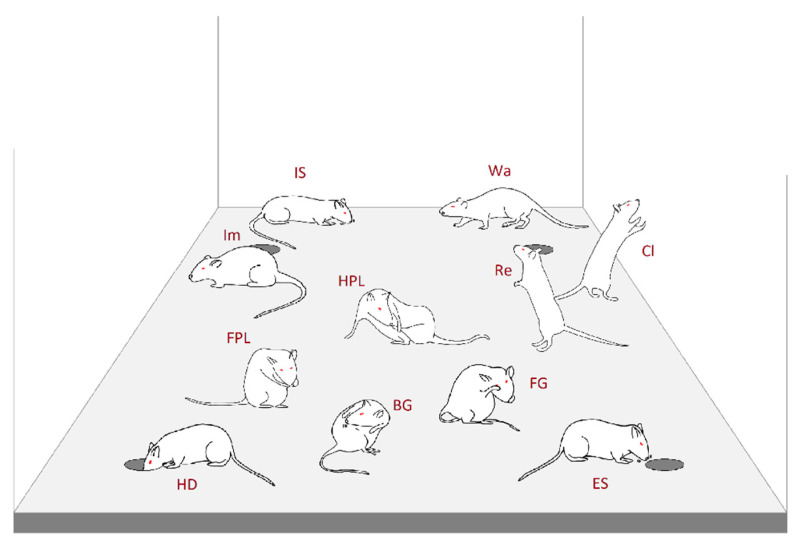
Behavioral repertoire of rat in the HB apparatus. Walking (**Wa**): rat walks around sniffing the environment; Immobile-Sniffing (**IS**): rat sniffs the environment standing on the ground; Climbing (**Cl**): rat maintains an erect posture leaning against the Plexiglass wall; Rearing (**Re**): rat maintains an erect posture without leaning against the Plexiglass box; Immobility (**Im**): rat maintains a fixed posture and no movements are produced; Front-Paw Licking (**FPL**): rat licks or grooms its forepaws; Hind-Paw Licking (**HPL**): rat licks or grooms its hind paws; Face Grooming (**FG**): rat rubs its face (ears, mouth, vibrissae, eyes) with rapid circular movements of its forepaws; Body Grooming (**BG**): rat licks its body combing its fur with fast movements of incisors; Edge-Sniff (**ES**): rat sniffs the border of one of the four holes; Head-Dip (**HD**): rat puts its head into one of the four holes.

The HB consisted of a 50 × 50 cm arena made of white opaque Plexiglass with a raised floor with four equidistant holes 4 cm in diameter (Figure 1). Such an HB arena was surrounded by three white opaque Plexiglass walls and a front transparent one. All surrounding walls were 50 × 50 cm. A digital video camera (Toshiba HD-DV camcorder P10, Tokyo, Japan) was placed in front of the transparent wall. After experimental/recording sessions, video files of each subject were stored on a PC for the following analyses.

### 2.2. Animals and Drugs

Thirty male Wistar rats (Harlan Laboratories, Udine, Italy), 2 months old, weighing 250 ± 50 g, were used. The animals were housed in a room with a temperature of 23 ± 1 °C with the light on at 7:00 a.m. and off at 7:00 p.m. Standard laboratory pellets and water were freely accessible. On the testing day, rats were randomly assigned to three groups, each group encompassing 10 subjects. One group received 1 mL of saline intraperitoneally (IP) injected as the control; the second group was IP injected with diazepam (Roche, Milan, Italy) at the dose of 2 mg/kg dissolved in 1 mL of saline; finally, the last group was IP injected with beta-carboline-3-carboxylic acid-N-methylamide (FG7142) (Sigma-Aldrich, St. Louis, MO, USA), at the dose of 8 mg/kg dissolved in 1 mL of saline [40,41]. Diazepam and FG7142 doses used in this experiment were chosen since they have been demonstrated to induce, respectively, clear-cut anxiolytic and anxiogenic effects [8,9]. 

### 2.3. Procedure 

On the testing day, the rats were transferred to a testing room within their home cages and allowed to acclimate for 30 min far from the observational apparatus. The temperature in the testing room was maintained equal to the temperature in the housing room. For all subjects/groups, the time elapsed from injections to the beginning of the observations was 20 min. Each subject, experimentally naïve, was placed in the HB and allowed to freely explore the environment for 10 min. Each subject was observed only once. After each observation, the apparatus was carefully cleaned with ethyl alcohol (70%). 

### 2.4. Data Analysis

#### 2.4.1. Ethogram

The ethogram (namely, the list of all the components of the behavioral repertoire and their formal description) used in the present study is shown in Figure 1. Such an ethogram is based on components and behavioral categories employed in preceding studies [8,9,36,38,39]. It encompasses four main categories and eleven behavioral components: (1) General Exploration, i.e., components of the behavioral repertoire aimed at the analysis of all environmental cues excluding the holes—Walking (Wa), Immobile Sniffing (IS), Climbing (Cl) and Rearing (Re); (2) Focused Exploration, i.e., behavioral components aimed at the analysis of cues exclusively related to the holes—Edge-Sniff (ES) and Head-Dip (HD); (3) Grooming Activity, i.e., self-cleaning activities not related to the environmental exploration—Front-Paw Licking (FPL), Hind-Paw Licking (HPL), Face Grooming (FG) and Body Grooming (BG); and (4) the last category contains only Immobility (Im), namely, the complete lack of movement and as such, is not classifiable in any of the previous categories. Based on this ethogram, video files of each subject were annotated by a highly trained observer blind to the treatments, using a professional software tool (The Observer, Noldus Information Technology bv, Wageningen, The Netherlands); event log files were generated for each subject. These files were then utilized to perform both quantitative and T-pattern analyses (TPA).

#### 2.4.2. Temporal Structure of Behavior 

TPA is a technique conceived to reveal the temporal architecture of behavior. Such a multivariate approach requires the utilization of a software known as Theme (PatternVision Ltd., Reykjavik, Iceland). Theme is able to reveal hidden sequences of events based on the detection of statistically significant constraints on the intervals separating them. In brief, given a distribution of events occurring within a T0-Tx observation period, an algorithm compares the distributions of each pair of events, e.g., “a” and “b”, searching for a temporal interval so that “a” is followed by “b” within such a time window. If/when such a condition is verified, a first-level T-pattern (i.e., a T-pattern containing only the (a b), is detected; then the first level temporal pattern is considered “a” or “b” for the detection of T-patterns of higher-order, e.g., ((a b) c)…((a b)(c d))…((a b)(c d) e) and so on, up to any level). Concepts, theories and procedures concerning the detection and analysis of T-patterns can be found in various works from our laboratory and in different papers from the creator of this multivariate approach [10,11,12,13,14,15,16,17,18]. In the present study, to maintain results of TPA comparable with previous ones, we used the same rigorous detection parameters employed in previous studies [36,38,39], i.e., minimum occurrences = 6; significance level = 0.0001; lumping factor = 0.90; minimum percent of samples = 60.

#### 2.4.3. Statistics

Concerning conventional quantitative analyses, for each group, the following parameters of the behavioral response were analyzed: mean occurrences, mean durations and the ratio between Head-dip and Edge-Sniff, i.e., the evaluation of the relationship between these two behavioral components on the basis of their mean occurrences. For the T-pattern analysis, for each group we analyzed the following: the structure of all the different T-patterns (terminal strings), the length distribution of different T-patterns, both in real and randomly generated data; mean length of T-patterns, mean occurrences of T-patterns and, finally, percent distribution of T-patterns encompassing each behavioral component.

As for mean occurrences and mean durations of each component of the behavioral repertoire and the Head-Dip/Edge-Sniff ratio, possible significant results among groups were assessed using One-Way Analysis of Variance (ANOVA) followed by Newman–Keuls post-hoc test for multiple comparisons; *p* < 0.05 was considered a significant value.

As for TPA, even if each sequence is “fueled” by a significant relationship among the events, data with thousands of events could raise an important question, i.e., whether the T-patterns are detected only by chance. Theme software deals with such a critical issue by repeatedly randomizing and re-analyzing the original data, using the same search parameters utilized in the detection process performed in the real data. Then, the mean number of T-patterns of each length detected in the randomized data is compared with the number of patterns identified in the original data. Mean length and mean occurrences of T-patterns detected in real data were assessed using One-Way Analysis of Variance (ANOVA) followed by Newman–Keuls post-hoc test for multiple comparisons; *p* < 0.05 was considered a significant value. Finally, the percent distribution of T-patterns encompassing each component of the behavioral repertoire was assessed using the Chi-square test; *p* < 0.05 was considered significant.

## 3. Results

### 3.1. Quantitative Results

**Figure 2:**
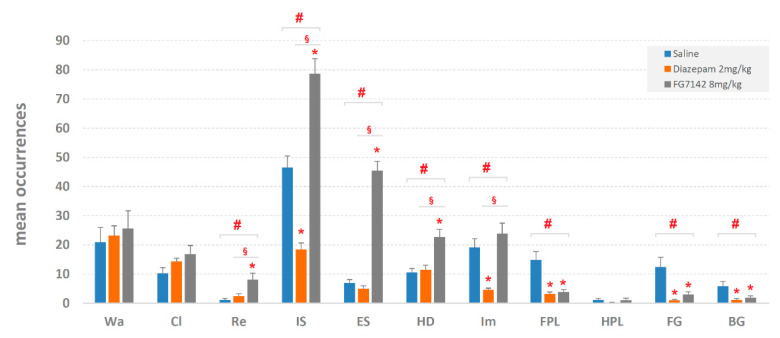
Mean occurrences ± SEM of each component of the behavioral repertoire (see Figure 1) in saline, diazepam 2 mg/kg and in FG7142 8 mg/kg groups. # = statistically significant (*p* < 0.05) ANOVA result; * = significant (*p* < 0.05) difference of diazepam or FG7142 groups in comparison with saline group (Newman–Keuls post-hoc test for multiple comparisons); § = significant (*p* < 0.05) difference between diazepam and FG7142 groups (Newman–Keuls post-hoc test for multiple comparisons). Data obtained from the analysis of three groups each encompassing 10 subjects. See Figure 1 for abbreviations.

Mean occurrences ± SEM of behavioral components are shown in Figure 2. ANOVA revealed significant results for Rearing (F_2,29_ = 7.07, *p* < 0.005), Immobile Sniffing (F_2,29_ = 57.41, *p* < 0.0001), Edge-Sniff (F_2,29_ = 125.42, *p* < 0.0001), Head-Dip (F_2,29_ = 12.43, *p* < 0.0001), Immobility (F_2,29_ = 14.30, *p* < 0.0001), Front-Paw Licking (F_2,29_ = 14.41, *p* < 0.0001), Face-Grooming (F_2,29_ = 9.21, *p* < 0.0001) and Body-Grooming (F_2,29_ = 5.43, *p* < 0.01). No significant results were detected for Walking (F_2,29_ = 0.22, *p* = 0.807), Climbing (F_2,29_ = 2.29, *p* = 0.120) and Hind-Paw Licking (F_2,29_ = 1.06, *p* = 0.359). The post-hoc analysis, performed by Newman–Keuls test, revealed several differences in comparison with saline, both for diazepam and FG7142. In addition, for various behavioral components, differences were detected between diazepam and FG7142 as well. Results of the post-hoc analysis for mean occurrences are illustrated in Figure 2.

**Figure 3:**
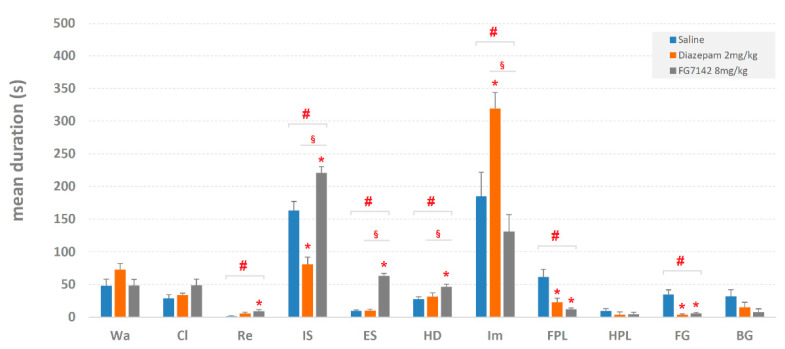
Mean duration ± SEM (in s) of each component of the behavioral repertoire (see Figure 1) in saline, diazepam 2 mg/kg and in FG7142 8 mg/kg groups. # = statistically significant (*p* < 0.05) ANOVA result; * = significant (*p* < 0.05) difference of diazepam or FG7142 groups in comparison with saline group (Newman–Keuls post-hoc test for multiple comparisons); § = significant (*p* < 0.05) difference between diazepam and FG7142 groups (Newman–Keuls post-hoc test for multiple comparisons). Data obtained from the analysis of three groups each encompassing 10 subjects. See Figure 1 for abbreviations.

Mean durations ± SEM (in sec) of behavioral components are shown in Figure 3. The ANOVA revealed significant results for Rearing (F_2,29 =_ 3.70, *p* < 0.05), Immobile Sniffing (F_2,29_ = 37.69, *p* < 0.0001), Edge-Sniff (F_2,29_ = 127.58, *p* < 0.0001), Head-Dip (F_2,29_ = 4.70, *p* < 0.05), Immobility (F_2,29_ = 10.67, *p* < 0.0001), Front-Paw Licking (F_2,29_ = 11.01, *p* < 0.0001) and Face Grooming (F_2,29_ = 17.52, *p* < 0.0001). No significant results have been detected for Walking (F_2,29_ = 2.09, *p* = 0.143), Climbing (F_2,29_ = 2.56, *p* = 0.096), Hind-Paw Licking (F_2,29_ = 0.62, *p* = 0.543) and Body Grooming (F_2,29 =_ 2.93, *p* = 0.071). The post-hoc analysis, performed by Newman–Keuls test, revealed several differences in comparison with saline both for diazepam and FG7142. In addition, for various behavioral components, differences were detected between diazepam and FG7142 as well. The results of the post-hoc analysis for mean durations are illustrated in Figure 3.

**Figure 4:**
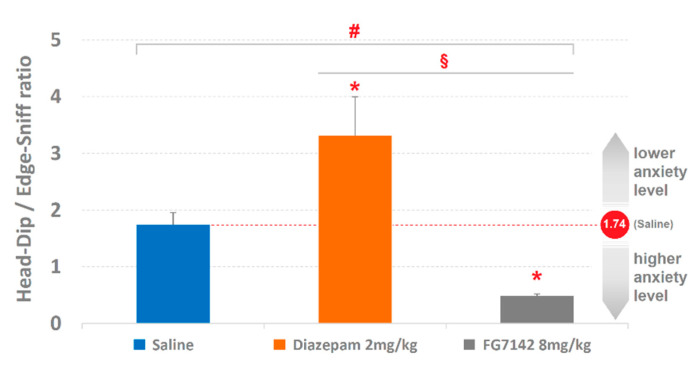
Effects of saline, diazepam 2 mg/kg and FG7142 8 mg/kg on the ratio between Head-Dip and Edge-Sniff. Data are represented as mean ± SE. # = statistically significant (*p* < 0.05) ANOVA result; * = significant (*p* < 0.05) difference of diazepam or FG7142 groups in comparison with saline group (Newman–Keuls post-hoc test for multiple comparisons); § = significant (*p* < 0.05) difference between diazepam and FG7142 groups (Newman–Keuls post-hoc test for multiple comparisons). Data obtained from the analysis of three groups each encompassing 10 subjects. See Figure 1 for abbreviations.

The ratio between Head-Dip and Edge-Sniff is presented in Figure 4. ANOVA revealed significant results (F_2,29 =_ 11.49, *p* < 0.0001). Newman–Keuls post-hoc test revealed significant (*p* < 0.05) differences in comparison with saline both for diazepam and FG7142; in addition, a significant (*p* < 0.05) difference was detected between diazepam and FG7142 as well.

### 3.2. Detection and Analysis of T-Patterns

**Figure 5:**
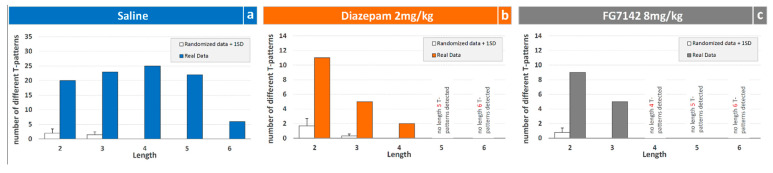
Overall number of different T-patterns (*y*-axis) detected based on their different length (*x*-axis) in rats injected with saline (**a**), diazepam 2 mg/kg (**b**) and FG7142 8 mg/kg (**c**). Filled bars = number of T-patterns detected in real data; white/empty bars = numbers of patterns +1SD detected in randomized data. Data obtained from the analysis of three groups each encompassing 10 subjects. See Figure 1 for abbreviations.

The distribution of T-patterns based on their length is illustrated in Figure 5. Overall, 96 different T-patterns were detected for saline, with 18 and 14 for diazepam and FG7142 groups, respectively. For each group, based on their length (that is, the number of events in sequence), T-patterns were distributed as follows. In the saline group, 20 different patterns encompass two events, 23 consist of three events, 25 four events, 22 five events and 6 T-patterns encompass six events in sequence (Figure 5a); as for the diazepam 2 mg/kg group, 11 different patterns encompass two events, 5 consist of three events and 2 patterns encompass four events in sequence (Figure 5b); finally, as for the FG7142 8 mg/kg group, 9 different patterns encompass two events and 5 T-patterns contain three events in sequence (Figure 5c). The randomization process, carried out to assess possible detections of T-patterns in randomized data, revealed for all groups a negligible amount of T-patterns detected in the random generated data (Figure 5, empty bars).

**Figure 6:**
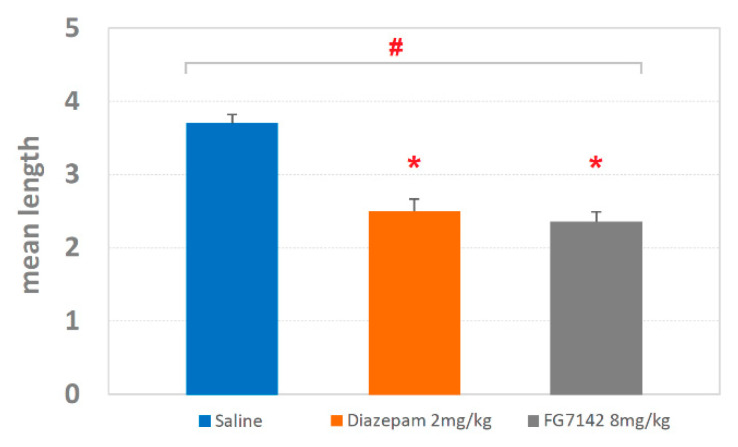
Mean length ± SEM of T-patterns detected in rats injected with saline, diazepam 2 mg/kg and FG7142 8 mg/kg. # = statistically significant (*p* < 0.05) ANOVA result; * = significant (*p* < 0.05) difference of diazepam or FG7142 groups in comparison with saline group (Newman–Keuls post-hoc test for multiple comparisons). Data obtained from the analysis of three groups each encompassing 10 subjects. See Figure 1 for abbreviations.

The mean length ± SEM of the T-patterns is shown in Figure 6. As for the saline group, the detected length was 3.69 ± 0.12; for diazepam, the length was 2.5 ± 0.16; finally, for FG7142, the detected length was 2.35 ± 0.13. The ANOVA revealed highly significant results (F_2,127_ = 16.54; *p* < 0.0001) with significant (*p* < 0.05) reduction for both diazepam and FG7142 in comparison with saline, as revealed by Newman–Keuls post-hoc test for multiple comparisons. 

**Figure 7:**
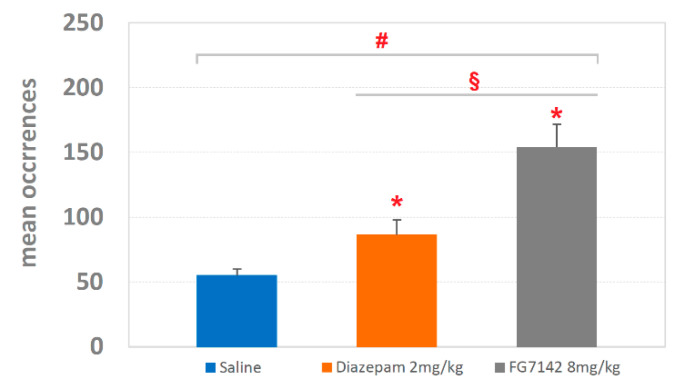
Mean occurrences ± SEM of T-patterns detected in rats injected with saline, diazepam 2 mg/kg and FG7142 8 mg/kg. # = statistically significant (*p* < 0.05) ANOVA result; * = significant (*p* < 0.05) difference of diazepam or FG7142 groups in comparison with saline group (Newman–Keuls post-hoc test for multiple comparisons); § = significant (*p* < 0.05) difference between diazepam and FG7142 groups (Newman–Keuls post-hoc test for multiple comparisons). Data obtained from the analysis of three groups each encompassing 10 subjects. See Figure 1 for abbreviations.

Mean occurrences ± SEM of T-patterns are shown in Figure 7. For the saline group, the detected occurrences were 54.97 ± 5.01; for diazepam, 86.38 ± 11.54; finally, for FG7142, occurrences were 153.92 ± 17.77. The ANOVA revealed highly significant results (F_2,127_ = 23.91; *p* < 0.0001) with significant (*p* < 0.05) increases for both diazepam and FG7142 in comparison with saline and a significant difference between diazepam and FG7142 as revealed by the Newman–Keuls post-hoc test for multiple comparisons. 

**Figure 8:**
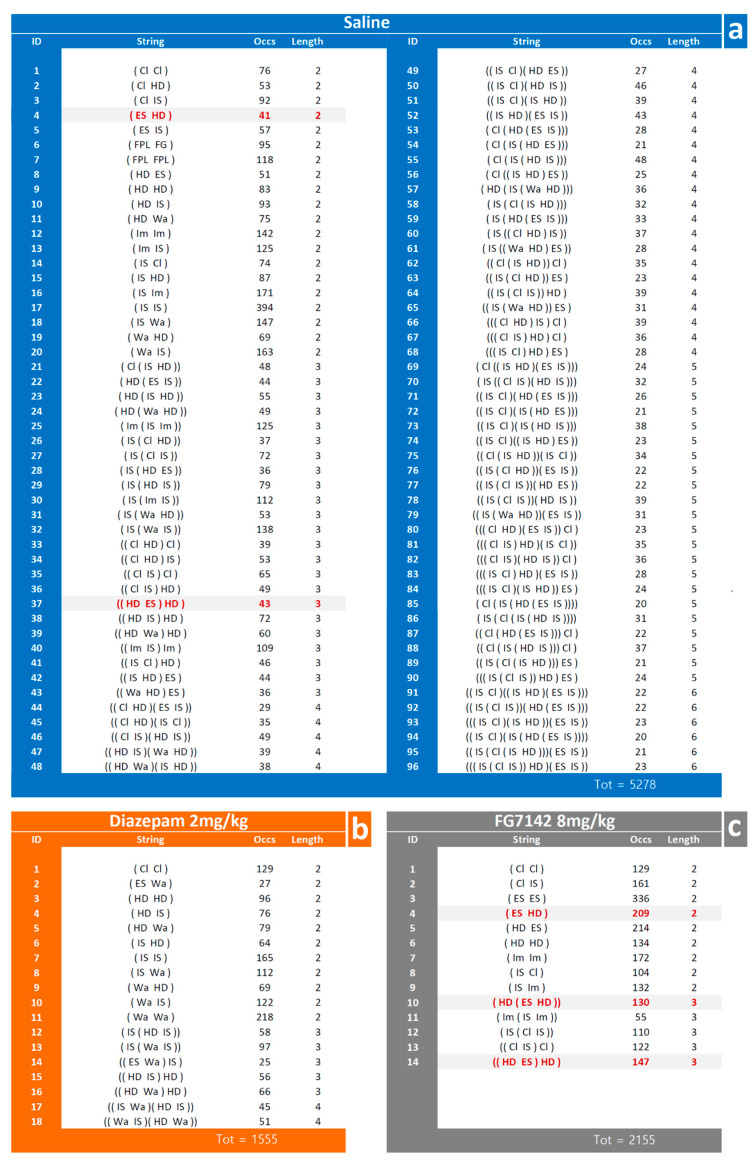
Terminal strings of the T-patterns detected in saline (**a**), diazepam 2 mg/kg (**b**) and FG7142 8 mg/kg (**c**) groups. The number on the left of each string indicate the corresponding T-pattern; numbers on the right of each string indicate their length and overall occurrences (Occs column). Highlighted in light grey: T-patterns containing Edge-Sniff → Head-Dip transitions. Data obtained from the analysis of three groups each encompassing 10 subjects. See Figure 1 for abbreviations.

The results of the T-pattern detection process in the saline, diazepam 2 mg/kg and FG7142 8 mg/kg groups are illustrated in Figure 8 in terms of terminal strings (that is, the textual representation of T-patterns detected). Numbers on the right of each string indicate their occurrences and length (Figure 8, “Occs” and “length” columns respectively). Overall, in the saline group, 96 different T-patterns occurred 5278 times (Figure 8a, “Occs” column); in the diazepam group, 18 different T-patterns occurred 1555 times (Figure 8b, “Occs” column); finally, in the FG7142 group, 14 different T-patterns occurred 2155 times (Figure 8c, “Occs” column). 

Taking into consideration the T-patterns encompassing each component of the behavioral repertoire, the results are presented in Table 1 in terms of the total amount of T-patterns containing each component and the related percent over the total amount of T-patterns. For the diazepam group, in comparison with saline group, significant (*p* < 0.01) changes were detected for Walking, Climbing, Immobile-Sniffing, Edge-Sniff and Head-Dip; for the FG7142 group, in comparison with the saline group, significant (*p* < 0.01) changes were detected for Climbing, Immobile-Sniffing, Edge-Sniff, Head-Dip and for T-patterns containing Edge-Sniff → Head-Dip transitions.

## 4. Discussion

In this study, alongside with conventional quantitative approaches, the Head-Dip/Edge-Sniff ratio was evaluated, and a T-pattern analysis was conducted to evaluate how Head-Dip and Edge-Sniff components are contextualized in the behavior structure. 

The main findings suggest that, under different emotional levels obtained using diazepam and FG7142, quantitative evaluations of isolated behavioral components are able to provide non-exhaustive, often misunderstood information. On the other hand, when the relationships between components of the behavioral repertoire are assessed, new information emerges, and new features of the rodent’s anxiety-related behavior become evident. 

### 4.1. Quantitative Analyses

Overall, even if diazepam and FG7142 operate on opposite sides of the subject’s emotional assets (respectively reducing or increasing the anxiety level), it is not possible to identify changes particularly evocative of modifications of the underlying anxiety level (Figure 2 and Figure 3). Indeed, some behavioral elements, i.e., Walking, Climbing and Hind-Paw Licking, do not change at all; in only one case, i.e., for Immobile-Sniffing, the changes occur in opposite directions. Other behavioral elements show, in comparison to the control, consensual reductions in their values but no difference between them, as occurs, for example, with various grooming behaviors. Interestingly, comparing to the control, one of the two drugs shows evident changes, while no variations are observed to be provoked by the other one: this is what happens in the FG7142 group for Rearing, Edge-Sniff and Head-Dip and in the diazepam group for Immobility. If, on the one hand, the above portrait demonstrates that, overall, the two drugs have a clear effect in behavioral terms, on the other hand, it raises a serious concern: the impossibility to coherently interpret these data in terms of anxiety-related behaviors. Such a difficulty is further amplified if, in detail, we consider the specific hole-exploration behaviors classically considered to mirror the emotional condition of the animal, i.e., Head-Dip and Edge-Sniff [8,38,39] and the existing approach–avoidance conflict, i.e., the internal conflict that the rodent must face between its natural tendency to explore the environment and, at the same time, to avoid possible dangers [42]. Counterintuitively, indeed, in the group treated with diazepam, these two behavioral elements do not change, neither in terms of occurrences nor in terms of duration. Even so, following the principle of approach–avoidance conflict, bearing in mind that diazepam acts in an anxiolytic sense, at least a considerable Head-Dip increase should be observed. As for the FG7142, on the contrary, evident increases in Head-Dip and Edge-Sniff are present. Again, based on the approach–avoidance conflict, since FG7142 is a powerful anxiety-inducing molecule, considerable reductions in these two parameters should be present. These counterintuitive data find relevant confirmations; for example, following the administration of diazepam, increases [43], decreases [44], or no modifications [8] of various Head-Dip parameters are reported. Conflicting evidence is also present for the FG7142 since Head-Dipping is reported to be decreased [43] or increased [9]. The conclusion can only move in one direction: to study anxiety-related behavior and, importantly, to evaluate the impact that changes in the central level of anxiety have, the quantitative evaluations of individual parameters, disjointed from the comprehensive structure of behavior (namely, the set of all the components of the behavioral repertoire and their relationships), may be a source of misunderstandings and conflicting results. It is not surprising nor unknown that behavioral research studies in the field of anxiety, during the last twenty years or so, have been, to some extent, very conservative in their techniques and analytical approaches. On this subject, an unfortunate and chronic association between the lack of converging findings and fresh approaches in the study of important neurological disorders, such as anxiety and depression, has been already underlined [45]. Quantitative evaluations, such as frequencies, durations, etc., of individual behavioral components should be partnered, whenever possible, with suitable approaches of behavioral analyses able to describe structural features of the observed behavior.

### 4.2. The Head-Dip/Edge-Sniff Ratio

To evaluate anxiety-related behaviors and the effects induced by various psychoactive substances, the situation considerably improves when the Head-Dip is related to the Edge-Sniff, and in particular, when the Head-Dip/Edge-Sniff ratio is assessed (Figure 4). The present results are well in agreement with previous findings [8,9] and show how the utilization of diazepam and FG7142 drives the ratio toward two opposite directions, respectively indicating the reductions and increases in the anxiety level. The explanation lies in the strong emotional value of the relationship linking these two behaviors of hole-exploration: Edge-Sniff is a behavior in which the animal sniffs the edge of the hole; Head-Dip occurs when the animal inserts its head into the hole. Of course, if the animal, after sniffing the edge, puts its head inside, this depends on the motivational drive, which in turn is greatly influenced by modifications of the emotional condition. So, if the animal often performs Head-Dip without a propaedeutic Edge-Sniff, then the resulting Head-Dip/Edge-Sniff ratio will increase due to an increase in the numerator: this is what occurs as a result of the reduction in the level of anxiety, for example, following diazepam [8]. On the contrary, if many Edge-Sniffs are not followed by as many Head-Dips, then the resulting Head-Dip/Edge-Sniff ratio will drop as a result of the increase in the denominator: this is what occurs following the increase in the level of anxiety, for example, following FG7142 [9]. The situation becomes even clearer and with less nuanced margins when a TPA is performed, and the Head-Dip and Edge-Sniff behaviors are contextualized in the more general behavioral architecture. Using the detection and analysis of T-patterns, indeed, it is possible to appreciate qualitative behavioral features otherwise impossible to detect by means of more conventional analyses. As a matter of fact, a synergic utilization of quantitative and qualitative approaches in a behavioral research study is able to provide a more comprehensive description of observed phenomena [27,46,47] greatly beyond what the human eye can intuitively detect.

### 4.3. T-Patterns

Both diazepam and FG7142 have an evident impact in terms of qualitative changes of T-patterns identified (Figure 5, Figure 6 and Figure 7). On this subject, it is worth mentioning that T-patterns are represented by three main qualitative features: (i) variability (i.e., the number of T-patterns of different composition), (ii) complexity (i.e., the number of events in T-pattern’s sequence) and, finally, (iii) recursivity (i.e., the number of times each T-pattern occurs) [27,39]. Simply stated, our data indicate that changes in emotional assets can dramatically modify the behavioral architecture. Figure 8 illustrates all the T-patterns of different compositions found in the three groups, how often they occur and their length. Table 1 presents the contents of this figure in a synoptic way, summarizing the presence of each behavioral event in T-patterns as overall occurrences and as a percentage. The two drugs induce considerable variations in different behavioral events, which increase or decrease when compared to the control group. Climbing and Immobile-Sniffing are noticeably less represented in both treated groups. All grooming and rearing behaviors are not included in the T-pattern. It is evident that from none of these events is it possible to draw conclusions about the emotional state of the subject since these results refer to the administration of molecules very well known for their activity in opposite directions, i.e., anxiolytic (diazepam) and anxiogenic (FG7142). On the other hand, outcomes concerning Head-Dip, Edge-Sniff and Walking highlight several interesting changes worthy of consideration. Head-Dip is a peculiar behavior in HB. The results indicate that, in percentage, it is reduced in all treated groups. The Edge-Sniff, on the contrary, is greatly reduced in rats injected with diazepam but considerably increased in subjects treated with FG7142 (Table 1). In other terms, in subjects with a reduced level of anxiety, the animal no longer structures Edge-Sniff in sequences of focused exploration and the Head-Dip, thus, remains lacking in the preparatory sniffing of the edge. It is possible to hypothesize that the reduction in the anxiety level becomes manifest, in behavioral terms, through the routing of behavioral sequences where the normal cautious approach to the exploration of the hole (from a causal point of view, a propaedeutic Edge-Sniff precedes the insertion of the head inside) almost disappears. Of course, in subjects with an increased anxiety level, an opposite situation would occur because the inclusion of Edge-Sniff in patterns is more than two-fold increased, in comparison with the control (Table 1). Consistently, when looking for T-patterns containing Edge-Sniff→ Head-Dip, it is easy to notice that they disappear in animals with a reduced level of anxiety; on the contrary, they reach almost 25% in animals with an increased level of anxiety. The peculiar “behavior” of Walking in the two treated groups deserves some further consideration. Walking is a generalized exploration activity [38,39] and very well represented in terms of mean occurrences in all groups (Figure 2). So, how is it possible that Walking is consistently increased in the T-patterns of the diazepam group, while it disappears from the sequences of the FG7142 group? We suggest that the reduction in the anxiety level further releases environmental exploration from the constraints imposed by normal cautious exploration. This would become manifest through greater inclusion of Walking in exploratory sequences. On the contrary, when the animal has a high level of anxiety, as in subjects treated with FG7142, the environmental exploration behavior becomes very cautious. This would result in a disappearance of walking from all exploration sequences and in the redirection of these exploration sequences toward the hole, which is an unknown object in the HB. Accordingly, the exploration of the hole remains very cautious, as underlined by the highly significant increase in Edge-Sniffs, in sequence. These findings strongly suggest that useful indicators of the rodent’s emotional level may be identified in Head-Dip, Edge-Sniff and Walking events, only when their patterning in behavioral sequences is evaluated.

## 5. Conclusions

It is well known that diazepam and FG7141, respectively an anxiolytic and an anxiogenic drug, modify anxiety levels. The anxiety condition, in turn, strongly influences the ratio of Edge-Sniff/Head Dip, evaluated by analyzing animal behavior in the Hole-Board test. A temporal pattern analysis reveals that Edge Sniff and Head-Dip are contextualized in the behavioral architecture and that Edge-Sniff → Head-Dip sequences are greatly influenced by the level of anxiety: barely detectable in control animals, these sequences disappear in subjects with a reduced level of anxiety and are present in almost the 25% of the T-patterns detected in subjects with increased anxiety. In addition, the present study claims that simple quantitative evaluations of individual behavioral elements, detached from the actual structure of behavior, can provide only fragmentary, and often not exhaustive, information on the subject’s anxiety-related behavior. On the contrary, when the relationships between the various elements of behavior are evaluated, otherwise undetectable information emerges that is strongly indicative of the individual’s emotional condition. Such an aspect begins to take shape from the simple bivariate relationship between Head-Dip and Edge-Sniff and becomes even more evident when the whole behavioral structure of the subject is taken into consideration.

## Figures and Tables

**Table 1:** Overall number and percent of T-patterns encompassing each behavioral event.

	Saline		Diazepam				FG7142			
ETP	Occs	%	Occs	%	χ^2^	Vs	Occs	%	χ^2^	Vs
Wa	993	18.81	911	58.59	<0.01	↑	0	0		↓
Cl	2033	38.52	129	8.30	<0.01	↓	626	29.05	<0.01	↓
IS	4248	80.49	871	56.01	<0.01	↓	684	31.74	<0.01	↓
Re	0	0	0	0		─	0	0		─
ES	1130	21.41	52	3.34	<0.01	↓	1036	48.07	<0.01	↑
HD	3003	56.90	660	42.44	<0.01	↓	834	38.70	<0.01	↓
ES→HD	84	1.59	0	0		↓	486	22.50	<0.01	↑
FPL	213	4.04	0	0		↓	0	0		↓
HPL	0	0	0	0		─	0	0		─
FG	95	1.80	0	0		↓	0	0		↓
BG	0	0	0	0		─	0	0		─
Im	910	17.24	0	0		↓	359	16.67	0.54	─

**ETP** column: name of the event encompassed in the structure of T-patterns; **Occs** column: total amount of T-patterns containing the given event; **%** column: percent value over the total amount of T-patterns detected in the group; **χ^2^** column: result of Chi-square test in comparison with sa-line group; vs. column: percent value, in comparison with saline, increased (↑), decreased (↓) or unchanged (─). Data obtained from the analysis of three groups each encompassing 10 subjects. See Figure 1 for abbreviations.

## Data Availability

The data presented in this study are available on request from the corresponding author.
